# The Bacteriohopanepolyol Inventory of Novel Aerobic Methane Oxidising Bacteria Reveals New Biomarker Signatures of Aerobic Methanotrophy in Marine Systems

**DOI:** 10.1371/journal.pone.0165635

**Published:** 2016-11-08

**Authors:** Darci Rush, Kate A. Osborne, Daniel Birgel, Andreas Kappler, Hisako Hirayama, Jörn Peckmann, Simon W. Poulton, Julia C. Nickel, Kai Mangelsdorf, Marina Kalyuzhnaya, Frances R. Sidgwick, Helen M. Talbot

**Affiliations:** 1 School of Civil Engineering & Geosciences, Newcastle University, Drummond Building, Newcastle upon Tyne, NE1 7RU, Newcastle-upon-Tyne, United Kingdom; 2 Institute of Geology, University of Hamburg, Hamburg, Germany; 3 Center for Applied Geoscience, University of Tübingen, Tübingen, Germany; 4 Center for Geomicrobiology, Department of Bioscience, Ny Munkegade 116, 8000, Aarhus C, Denmark; 5 Department of Subsurface Geobiological Analysis and Research, Japan Agency for Marine-Earth Science & Technology (JAMSTEC), Yokosuka, Japan; 6 Department of Geodynamics and Sedimentology, University of Vienna, 1090, Vienna, Austria; 7 School of Earth and Environment, University of Leeds, Leeds, LS2 9JT, United Kingdom; 8 GFZ German Research Centre for Geosciences, Telegrafenberg, D-14473, Potsdam, Germany; 9 Faculty of Biology, San Diego State University, 5500 Campanile Drive, San Diego, 92182, United States of America; Fudan University, CHINA

## Abstract

Aerobic methane oxidation (AMO) is one of the primary biologic pathways regulating the amount of methane (CH_4_) released into the environment. AMO acts as a sink of CH_4_, converting it into carbon dioxide before it reaches the atmosphere. It is of interest for (paleo)climate and carbon cycling studies to identify lipid biomarkers that can be used to trace AMO events, especially at times when the role of methane in the carbon cycle was more pronounced than today. AMO bacteria are known to synthesise bacteriohopanepolyol (BHP) lipids. Preliminary evidence pointed towards 35-aminobacteriohopane-30,31,32,33,34-pentol (aminopentol) being a characteristic biomarker for Type I methanotrophs. Here, the BHP compositions were examined for species of the recently described novel Type I methanotroph bacterial genera *Methylomarinum* and *Methylomarinovum*, as well as for a novel species of a Type I *Methylomicrobium*. Aminopentol was the most abundant BHP only in *Methylomarinovum caldicuralii*, while *Methylomicrobium* did not produce aminopentol at all. In addition to the expected regular aminotriol and aminotetrol BHPs, novel structures tentatively identified as methylcarbamate lipids related to C-35 amino-BHPs (MC-BHPs) were found to be synthesised in significant amounts by some AMO cultures. Subsequently, sediments and authigenic carbonates from methane-influenced marine environments were analysed. Most samples also did not contain significant amounts of aminopentol, indicating that aminopentol is not a useful biomarker for marine aerobic methanotophic bacteria. However, the BHP composition of the marine samples do point toward the novel MC-BHPs components being potential new biomarkers for AMO.

## Introduction

Methane (CH_4_) is a potent greenhouse gas, and its atmospheric concentration has tripled since pre-industrial times (e.g. [1,2]). Global oceans hold large subsurface reservoirs of CH_4_ in the form of gas hydrates. These stores are precariously dependent on temperature and pressure. A rapid destabilisation of gas hydrates has been proposed to have caused vast releases of marine CH_4_ in the past [3]. Increased input of CH_4_ into the atmosphere has been interpreted through records of excursions of significant δ^13^C depletion in the geological record, such as in the Palaeocene-Eocene Thermal Maximum (PETM) [4–7].

CH_4_ release into the atmosphere is regulated by methanotrophy, which converts CH_4_ into CO_2_, thereby playing a key role in the carbon biogeochemical cycle [8]. Although traditionally anaerobic archaea have been the most studied methanotrophs (e.g., AMNE-1 and ANME-2; cf. [9]), recent observations have highlighted the importance of bacteria performing aerobic CH_4_ oxidation (AMO) in marine, estuarine, and riverine fan environments (e.g., [10–14]). For example, pelagic AMO activity rose significantly after the Macondo oil well blowout in 2010 [15]. However, this activity was short-lived, highlighting the complexity of natural community interactions in response to increased CH_4_ [16]. It is thus important to recognise and trace methanotrophy during past extreme events in order to understand its potential to mitigate future CH_4_ release.

AMO bacteria belong to two phyla, *Proteobacteria* and *Verrucomicrobia*. Most isolates of *Verrucomicrobia* are thermoacidophilic [17–20], and have been found primarily in acidic, geothermal environments [21]. Aerobic methanotrophic members of *Proteobacteria* belong to two distinct classes, separated based on their carbon assimilation pathways [8]. Type II methanotrophs, members of the *Alphaproteobacteria*, are associated with terrestrial settings ([8], and references therein), whereas Type I methanotrophs members of the *Gammaproteobacteria* are widespread in aquatic systems, although they are also found in terrestrial systems. Both Type I and Type II methanotrophs are known to synthesise bacteriohopanepolyol (BHP) lipids [22]. BHPs are precursors to hopanes, which are the most ubiquitous lipids in the geological record [23,24]. Therefore, being able to trace AMO using hopanoid biomarkers is of value to the study of the carbon cycle in the past.

**Fig 1:**
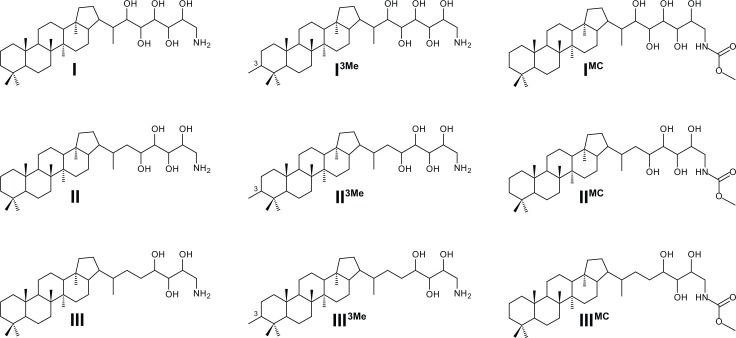
Chemical structures of bacteriohopanepolyol lipids. **I,** aminopentol; **I**^**3Me**^, 3-methyl-aminopentol; **I**^**MC**^, methylcarbamate-aminopentol; **II**, aminotetrol; **II**^**3Me**^, 3-methyl-aminotetrol; **II**^**MC**^, methylcarbamate-aminotetrol; **III**, aminotriol; **III**^**3Me**^, 3-methyl-aminotriol; **II**^**MC**^, methylcarbamate-aminotriol. The proposed structure of methylcarbamate(MC)-aminopentol (**I**^**MC**^), MC-aminotetrol (**II**^**MC**^), and MC-aminotriol (**III**^**MC**^) are tentatively based on mass spectral identification (S1 File).

Previous work exploring hopanoids as biomarker lipids found that methylation at the C-3 position (3-Me-BHPs; Fig 1, **I**^**3Me**^, **II**^**3Me**^, **III**^**3Me**^) was a possible indication of methanotrophic origin [25,26]. However, attributing C-3 methylation to AMO was challenged by the revelation that bacteria other than methanotrophs have the genes to methylate at C-3 [27]. C-3 methylation is more likely a requirement for cell survival in late stationary phase [27]. Moreover, not all methanotrophic bacteria have the gene to methylate at this position, [27,28], nor are 3-Me BHP precursors found in all CH_4_-influenced environments (Table 1). However, AMO bacteria are often considered to be the most likely source of 3-Me hopanoids in marine sediments due to their depleted carbon isotope signatures (e.g., [29]) and because they are frequently accompanied by 4-methylated steroids, which are also known as biomarkers of methanotrophic bacteria (e.g., [30,31]). In other studies, diplopterol and diploptene have been interpreted as biomarkers for methanotrophy, notably in anoxic environments [32–35]. However, neither diplopterol nor diploptene is source-specific to AMO [36], and these studies also relied on very negative δ^13^C lipid values (e.g., ca. -61‰ for diplopterol; -61 to -74‰ for diploptene) as an indication of CH_4_ being the carbon source for the organism producing these lipids. Yet, recent work has emphasised that not all AMO-derived carbon shows a depleted isotope signature, especially in terrestrial systems where Type II methanotrophs tend to dominate. For example, only limited depletion in ^13^C, with values between -25‰ and -40‰, have been reported for hopanoids with an inferred methanotrophic origin from modern peat bogs [37,38] as well as other ancient lignite deposits [39]. Also, BHPs in Congo deep sea fan sediments, originating from low-latitude wetlands [40], had higher ^13^C values than expected (i.e., C_30_ hopanol ~ -41‰; [14]). In marine CH_4_ seep carbonates from the Gulf of Mexico, BHPs, hopanoic acids, and 4-methylated sterols of aerobic methanotrophs were found with similar δ^13^C values as the CH_4_ source [41]. These observations can be the result of dilution from other heterotrophic bacterial sources that make it difficult to identify subordinate methanotroph contributions [37]. The metabolic pathways used by the AMO bacteria for CH_4_ assimilation can also have a profound effect on the level of isotopic depletion, with values for serine pathway methanotrophs (Type II) ranging from 12‰ depleted to 10‰ enriched relative to the CH_4_ substrate [42]. Furthermore, to analyse δ^13^C of intact BHPs, these must first be converted into primary alcohols by periodic acid/sodium borohydride cleavage [43]. For example, the δ^13^C value of 35-aminobacteriohopane-30,31,32,33,34-pentol (aminopentol; Fig 1, **I**) is measured on the C_30_-hopanol product, which includes all converted hexa-functionalised BHPs (i.e., not only aminopentol), as well as any free-hopanols that are present in a sample before BHP conversion. While the contamination of free-hopanols can be circumnavigated by column separations (e.g., [41]), measuring the δ^13^C values of intact BHPs is not currently possible.

**Table 1:** Presence and absence of aminopentol and related methylated and unsaturated homologues in previously investigated environmental settings.

	aminopentol			aminotetrol		aminotriol		
Sample	I	ΔI	I^3Me^	II	II^3Me^	III	III^3Me^	Reference
**Soils**								
Pasture [manured] (UK)	+	-	-	+	-	+	+	[112]
Pasture [not manured] (UK)	-	-	-	+	-	+	+	[112]
Rice Paddy (Vietnam)	+	-	-	+	-	+	-	[112]
Woodland (North East England)	+	-	-	+	-	+	+	[112]
South West France	+	-	-	+	-	+	-	[116]
Amazon	+	-	-	+	-	+	-	[109]
Congo	+	+	-	+	-	+	+	[40]
Lean Delta Peamafrost [ice complex]	+	-	-	+	-	+	-	[110]
Human Sewage [treated]	-	-	-	+	-	+	-	[117]
Forest, Grassland Soils (Alberta, Canada)	-	-	-	+	-	+	-	[118]
**Peat and Lignite**								
River Tet Catchment (France)	+	-	-	+	-	+	-	[116]
Moorhouse (UK)	+	-	-	+	-	+	-	[46]
Misten Bog (Belgium)	+	-	-	+	-	+	-	[119]
Bisendorfer Moor (Germany)	+	-	-	+	-	+	+	[111]
The Cobham Lignite (UK)	+	-	-	+	-	+	-	[49]
**Geothermal Environments**						+		
Cyanobacterial mat (Surprise Valley, Nevada, USA)	+	-	-	+	-	+	-	[120]
Silica Sinter (Orakie Korako, Taupo Volcanic Zone, New Zealand)	+	-	+	+	-	+	-	[113]
Silica Sinter (Champagne Pool, Taupo Volcanic Zone, New Zealand)	-	-	-	-	-	+	-	[121]
Orange mat (Yellowstone, USA)	-	-	-	-	-	+	-	[122]
**Other Microbial mats**								
Mars Oasic (Antarctica)	-	-	-	-	-	+	-	[48]
Hypolith (Devon Island, Arctic)	-	-	-	-	-	+	-	[48]
Cyanobacterial Mat (Christmas Island, Kiribati)	-	-	-	-	-	+	-	[123]
**Lake Sediments**								
Lake Windermere (UK)	+	-	-	+	-	+	-	^¶^
Lake Cadagno (Switzerland)	+	-	-	+	-	+	-	[26]
Lake Holzmaar (Germany)	+	-	-	+	-	+	-	[26]
Lake Nkunga (Kenya)	+	-	-	+	-	+	-	[45]
Priest Pot (England)	+	-	-	+	-	+	-	[26,45]
La Piscina de Yuriria (Mexico)	+	-	+	+	+	+	-	[26,45]
Laguna de Zempoala (Mexico)	+	-	-	+	-	+	-	[26]
Loch Ness (UK)	+	-	-	+	-	+	-	[26,45]
Lake Druzhby (Antarctica)	+	-	-	+	-	+	-	[26,45]
Sombre Lake (Signy Island, Southern Atlantic Ocean)	+	-	-	+	-	+	-	[26]
Heywood Lake (Signy Island, Southern Atlantic Ocean)	+	-	-	+	-	+	-	[26]
Ace Lake [Freshwater unit]	+	-	-	+	-	+	-	[104]
Ace Lake [meromictic unit]	-	-	-	+	-	+	-	[104]
Ace Lake [marine unit]	-	-	-	-	-	+	-	[104]
**Wetlands**								
Amazon	+	+	-	+	-	+	-	[109]
Congo	+	+	-	+	-	+	-	[40]
**Stream/River/Estuary Sediments**								
Arctic Rivers (Indigirka, Kolyma, Lena, Ob, Yenisei, Yukon, Mackenzie)	+	-	-	+	-	+	-	[124]
Glacial Outflow Stream (Svalbard)	+	-	-	+	-	+	-	[125]
Kalix River [surface sediment transect]	+	-	-	+	-	+	-	[126]
Kolyma River [surface sediment transect]	+	-	-	+	-	+	-	[127]
Yenisei River [mouth, and surrounding area]	+	-	-	+	-	+	-	[128]
Yangtze River [estuary, inner shelf]	+	-	-	+	-	+	-	[82,129]
Congo River [estuary]	+	+	-	+	-	+	-	[14,40]
**Water Column**								
Priest Pot [pond] (UK)	+	-	-	+	-	+	-	^¶^
River Water (Panama)	+	-	-	+	-	+	-	[130]
Yenisei River	+	-	-	+	-	+	-	[128]
Black Sea [oxic/anoxic transition zone]	+	-	+	+	-	+	-	[12]
Black Sea [oxic/anoxic transition zone]	+	-	-	+	-	+	-	[108]
Gotland Deep, Baltic Sea	+	-	-	+	-	+	-	[107]
Marine Water (off Panama coast)	-	-	-	-	-	+	-	[130]
Pelagic [sub-oxic and anoxic] (Arabian Sea, Peru Margin, Cariaco Basin)	-	-	-	-	-	+	-	[90]
Cariaco Basin	-	-	-	-	-	+	-	[131]
California Current	-	-	-	-	-	+	-	[91]
**Modern/Recent Marine Sediments**								
Gotland Deep, Baltic Sea	+	-	-	+	-	+	-	[107]
Congo River Deep-Sea Fan	+	+	-	+	-	+	-	[14]
Amazon Shelf and Fan	+	-	-	+	-	+	-	[109]
East Siberian Arctic Shelf	+	-	-	+	-	+	-	[79,126]
Black Sea	-	-	-	+	-	+	-	[88]
Southwest African Coast	-	-	-	+	-	+	(+)	[89]
Chukchi Sea	-	-	-	+	-	+	-	[132]
Alaskan Beaufort Sea	-	-	-	-	-	+	-	[132]
Baltic Sea	-	-	-	+	-	+	-	[133]
**Marine Carbonates**								
Carbonate, Gulf of Mexico	-	-	-	+	-	+	-	[72]
Authigenic carbonates, Gulf of Mexico	-	-	-	+	-	+	-	[41]
Seep Carbonate, Arabian Sea	-	-	-	+	-	+	-	[106]
**Other samples**								
Membrane Foulant [river water]	+	-	-	-	-	+	-	[134]
Membrane Foulant [brackish water]	+	-	-	+	-	+	-	[134]
Membrane Foulants [seawater]	-	-	-	-	-	+	-	[134]
Mussel Gill Tissue	-	-	-	+	-	+	-	[103]

+, detected in at least one of the samples; -, not detected in any of the samples

(+) indicates a methylated aminotriol was detected but the position of methylation was not identified

¶Talbot and Farrimond, unpublished data

Aminopentol is thought to be the most diagnostic BHP for AMO (see review in [14]). Aminopentol and its methylated and unsaturated homologues (i.e., **I**^**3Me**^, **ΔI**) have been found almost exclusively in Type I aerobic methanotrophs [22,44–46]. Moreover, aminopentol (**I**) has been found in a wide range of environments, which indicates potential as a biomarker for AMO (Table 1). Additionally, 35-aminobacteriohopane-31,32,33,34-tetrol (aminotetrol, **II**) and 35-aminobacteriohopane-32,33,34-triol (aminotriol, **III**) are also synthesised by Type I and Type II AMO bacteria. However, **II** and **III** are less source-specific as both are synthesised by some species of sulfate reducing bacteria (SRB) of the genus *Desulfovibrio* [47], and **III** is synthesised by many other aerobic bacteria ([48], and references therein).

Aminopentol made up a very minor proportion of the BHP composition in a SRB culture (<0.1% of total BHPs in *Desulfovibrio salexigens*). The ratio of aminopentol to the more ubiquitous aminotriol was 1:1352 [47]. We can therefore discount SRB as the source of aminopentol in an environmental sample with a high ratio of aminopentol:aminotriol. A similar approach was recently used by [49] based on the ratio of aminotetrol:aminotriol which has been found in the range 1:20–100 in some species of *Desulfovibrio* SRB [47,50,51]. Interestingly, some of the species of SRB cultures analysed by Blumenberg et al. [47,50,51] also synthesised diplopterol and diploptene, which could explain the enhanced presence of these lipids in CH_4_-influenced anoxic sites.

Representatives from only a small number of Type I methanotroph genera have been tested for BHP production (e.g., [22,36,46,52,53]. Many more recently described genera, including novel genera from marine and other (hyper)saline environments, have yet to be explored (e.g., [54–56]). Moreover, relatively few studies of BHP distributions have targeted marine environments (Table 1). It is important, therefore, to determine whether aminopentol, which is seen as a diagnostic marker for Type I methanotrophs, is present in methanotrophs isolated from marine and other saline environments, and whether we can find aminopentol in CH_4_-influenced marine sediments. This knowledge will have implications for the use of aminopentol as a biomarker to trace AMO in modern and ancient marine environments.

In this study, our goal was to develop an appropriate biomarker approach for AMO, which will allow high throughput analysis of sediment without the requirement for laborious chemical conversion steps prior to compound specific isotope analysis. To this end, we screened the BHP distributions of three genera of aerobic methanotrophs (i.e., *Methylomicrobium*, *Methylomarinum* and *Methylomarinovum)*. *Methylomarinum* and *Methylomarinovum* have not previously been investigated for BHPs. Two species of *Methylomicrobium* have been described previously [52,53], however, we include an additional species *Methylomicrobium kenyense*. These data are combined with literature BHP distributions of other AMO genera, including the recently reported Type I genus, *Methylobacter* [28], in order to facilitate interpretation of BHP distributions in six selected modern marine sediments from CH_4_-influenced systems. Two marine sites not influenced by CH_4_ were also investigated as controls for background marine BHP signatures.

## Methods

### Methanotroph pure cultures

#### Cultivation of *Methylomarinum vadi* IT-4

Previously described *Methylomarinum vadi* IT-4 was isolated from a microbial mat sample (in-situ temperature 30–40°C) collected at a shallow marine hydrothermal system (depth, ~23 m) in a coral reef off Taketomi Island, Okinawa, Japan [56,57]. Cultivation of this strain was performed at JAMSTEC, Japan, using MJmet medium at pH 6.6 at 37°C. A detailed site description and the enrichment and isolation procedures can be found in [57].

#### Cultivation of *Methylomarinovum* spp

Previously described *Methylomarinovum caldicuralii* IT-9 was isolated from the hot vent fluid (52°C) collected at the main vent site (depth, 23 m) in the shallow marine hydrothermal system where *Methylomarinum vadi* IT-4 was isolated [55,57]. *Methylomarinovum* sp. IN45 is a new isolate from a deep-sea hydrothermal field in Okinawa Trough, Japan (H. Hirayama, pers. comm.). The cultivation of strains IT-9 and IN45 was performed at JAMSTEC, Japan, using MJmet medium at 45°C, and at pH 6.2 and 6.6, respectively.

#### Cultivation of *Methylomicrobium* spp

*Methylomicrobium alcaliphilum* and *Methylomicrobium kenyense* were first isolated from highly alkaline soda lakes in Russia and Kenya, respectively [58]. Both *M*. *alcaliphilum* (DSM-number 19304) and *M*. *kenyense* (DSM-number 19305) are from the Leibniz Institute DSMZ (German Collection of Microorganisms and Cell Cultures). The cultivation of both strains was done at the Center for Applied Geosciences at the University of Tübingen, Germany, at pH 9.1 and at 28°C with a high salt NMS medium (1.5% NaCl).

### Marine sediment and carbonate samples

#### Håkon Mosby Mud Volcano (HMMV)

HMMV has been extensively studied for both aerobic and anaerobic methanotrophy [13,59]. The flow of CH_4_ in the center of the HMMV is restricted, and AMO was observed to be the most dominant process within the centre of the crater, performed by Type I methanotrophs [60]. Sediments from the HMMV were collected aboard the *RV Polarstern* (cruise ARK XXIV/2; 2009) and the *RV Maria S Merian* (cruise MSM 16/2; 2010) [61].

#### Barents Sea carbonate crust (BSCC)

The Barents Sea is a well-studied area of active CH_4_ seepage. The seafloor geology is marked by pockmarks [62], gas hydrates, gas flares [63], and patches of carbonate crusts [64], indicating active CH_4_ seepage [10,64]. We analysed eight depths (each 2–3 cm thick) from a push core (P120020 PR4) of a cold seep carbonate crust sampled at Loppa High/Polheim Sub-Platform area in the SW Barents Sea (72° 34’02.07”N, 20° 52’05.96”E). The core penetrated to a depth of 19 cm below sea floor (cmbsf). These samples were taken aboard the *RV Fugro Meridian* in September 2012 by Lundin Petroleum Norway.

#### Amon Mud Volcano (AMV)

The Amon mud volcano (AMV) is located in the Nile deep-sea fan, in the Eastern Basin of the Mediterranean Sea. Hydrocarbons, muds, and fluids are transported to the surface via one main feeding channel from the deep subsurface, creating a stark thermal gradient in the sediment. Oxidation of CH_4_ in the water column directly above AMV has been attributed to AMO using ^13^C and ^2^H isotopic values [65]. Sediments from AMV were collected aboard the *RV Meteor* (cruise M70/2, BIONIL; 2006) and the *RV Maria S Merian* (cruise MSM 13/3; 2009) [66].

#### New Zealand Seeps (NZS)

The Hikurangi continental margin, east of New Zealand’s North Island has been described as a biogeographically “new” cold seep province, characterised by endemic faunal communities [67]. Surface (0–2 cmbsf) and subsurface (10–12 cmbsf) sediment samples from three New Zealand seep (NZS) sites were collected aboard the *RV Sonne* (cruise So-191; 2007) [68]. These were dominated by distinct biota: *Frenulata* (Omakere Ridge), *Ampharetidae* (Wairarapa Takahae), and sulfur-oxidising bacteria (Wairarapa Takahae).

#### Gulfo Dulce (GD) surface sediments

A sill at 60 m water depth physically cuts off Golfo Dulce from the Pacific Ocean, which promotes anoxic conditions within the basin. Recently, GD was shown to contain authigenic carbonate formations at shallow (ca. 10 m) water depth [69]. GD sediments were collected along a transect from 10 to 140 m water depth in March 2008, as described in [70].

#### Gulf of Mexico (GoM) cold seeps

The Gulf of Mexico (GoM) holds an abundance of thermogenic gas. The venting of this gas from deep subsurface forms gas hydrates, free CH_4_, which are the sources of energy for microbial chemosynthetic communities, and authigenic carbonates [71]. Pancost et al., [72] described the BHP composition of material from five carbonate rock and nodule sites in GoM. Two of the carbonates were shown to contain low quantities of aminopentol. Here, we reinvestigated the BHP signatures of these sediments.

#### GoM sediments

The GoM also houses the outflow of the Mississippi River Delta, transporting terrestrial material into the Gulf. Three GoM sites to the best of our knowledge not influenced by CH_4_ (27°30'N, 87°20'W; 28°20'N, 89°38'W; 26°50'N, 92°40'W; two sediment depths at each site) were investigated in this study [73].

#### Peru Margin (PM)

An intense upwelling regimes fertilises surface water productivity on the Peru Margin (PM). This lends to oxygen utilisation in the water column, causing the Eastern South Pacific Oxygen Minimum Zone (ESP OMZ) [74]. Three PM sediments were analysed (10–15, 20–25 and 40–45 cmbsf) from a core taken within the ESP OMZ, at 100 m water depth [73,75].

### Lipid extraction

#### Total lipid extraction

All freeze-dried bacterial cells and marine sediments, except the Barents Sea samples extracted at GFZ Potsdam, were extracted using a modified Bligh-Dyer method [76,77]. Briefly, freeze-dried material was extracted in 19 mL of a 10:5:4 (v:v:v) mixture of MeOH:chloroform:H_2_O in a 50 mLTeflon tube. This mixture was sonicated for 15 min at 40°C, and centrifuged for 10 min. The supernatant was transferred to a second tube, and the residue re-extracted twice more. The chloroform in the supernatant was separated from the aqueous phase by adding water until the H_2_O:MeOH ratio was 1:1 (v:v), and collected. This procedure was repeated for the subsequent extractions. The collected chloroform total lipid extract (TLE) was dried by rotary evaporation in a round-bottom flask. The extraction protocol at GFZ Potsdam was similar but used a mixture of MeOH:DCM:ammonium acetate buffer [78].

#### Solid Phase Extraction

In-house comparisons have shown that amino-BHPs are better detected after solid phase extraction (SPE). An aliquot of the TLE was separated over a 1 mg NH_2_ solid phase extraction cartridge, as described in [79]. Briefly, the aliquot was dissolved and loaded onto a hexane-rinsed cartridge using 200 μL chloroform. Six mL of a 98:2 (v:v) diethyl ether:acetic acid solution was eluted. Residual material was dissolved with 200 μL 2:1 (v:v) chloroform:MeOH and loaded onto the cartridge, followed by elution with 10 mL of MeOH. BHPs were isolated from the MeOH fraction.

### Lipid analyses

#### BHP preparation and HPLC/APCI–MSn analyses

A known amount (ca. 5–10 μg/g dry sediment) of internal standard (5α-pregnane-3β,20β-diol) was added to SPE extracts of the TLE for BHP analysis. Samples were acetylated in 0.5 mL of a 1:1 (v:v) mixture of acetic anhydride and pyridine at 50°C for 1 h, then left to stand overnight at room temperature [80]. Solvent was dried under a stream of N_2_ on a 50°C heating block. BHP samples were dissolved in MeOH:propan-2-ol (3:2; v:v), and filtered on 0.2 μm PTFE filters.

BHPs were analysed by high performance liquid chromatography coupled to positive ion atmospheric pressure chemical ionization mass spectrometry (HPLC/APCI-MS), using a data-dependent scan mode (2 events) on an HPLC system equipped with an ion trap MS, as described in [46,81]. Further structural information for novel BHPs was obtained by way of MS^3^ spectra. BHP concentrations were (semi) quantitatively estimated based on the response factor of authentic standards (M. Rohmer; Strasbourg, France and [46,77]) relative to the internal standard.

## Results

In this study we investigated the BHP distributions in species of three AMO marine genera, and of eight marine environments, six of which were CH_4_-influenced.

### Novel nitrogen-containing BHP components

**Fig 2:**
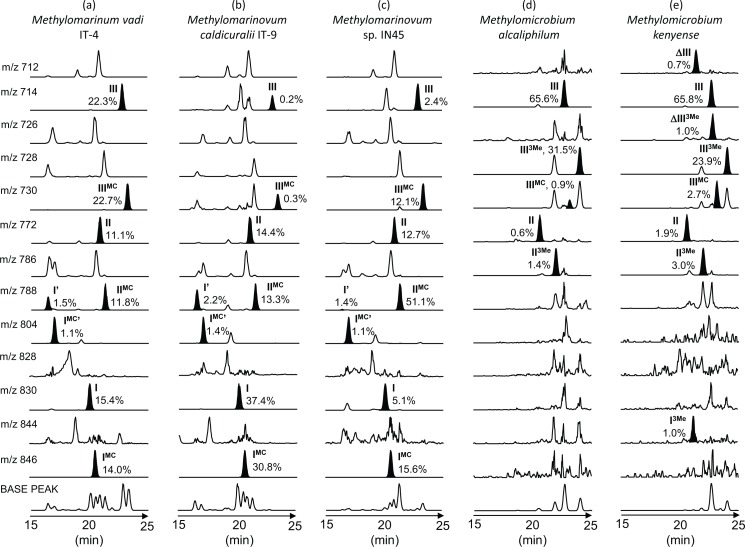
Distribution of nitrogen-containing BHPs in novel Type I methanotroph cultures. Partial mass chromatograms (15–25 min) showing relative abundances (%) of BHPs (shaded peaks) in the acetylated total lipid extracts of (a) *Methylomarinum vadi* IT-4, (b) *Methylomarinovum caldicuralii* IT-9, (c) *Methylomarinovum* sp. IN45, (d) *Methylomicrobium alcaliphilum*, and (e) *Methylomicrobium kenyense*.

In addition to the ‘regular amino-BHPs’ (e.g., **I**, **II**, and **III**; Figs 1 and 2), a suite of novel compounds were found in the methanotrophs and screened marine samples. Identification of these compounds is described in detail in the Supplementary Information (S1 File). Briefly, these components were related to the 35-amino-BHPs but differ in their terminal groups at C-35, which are tentatively proposed to comprise a methylcarbamate rather than a simple amine on the basis of interpretation of their APCI MS^2^ and MS^3^ spectra. In each case, the novel compounds (**I**^**MC**^, **I**^**MC**^**’**, **II**^**MC**^, **III**^**MC**^; Figs 1 and 2) elute after their ‘regular’ amino-BHP analogues (i.e., **I**, **I’**, **II**, **III**; Fig 2). This indicates that the tentatively-assigned terminal group structures are less polar than the regular terminal amines (after acetylation). The novel structures include: 35-methylcarbamate-bacteriohopane-32,33,34-triol (MC-triol herein; **III**^**MC**^), 35-methylcarbamate-bacteriohopane-31,32,33,34-tetrol (MC-tetrol herein; **II**^**MC**^), 35-methylcarbamate-bacteriohopane-30,31,32,33,34-pentol (MC-pentol herein; **I**^**MC**^) and an isomer of **I**^**MC**^ (**I**^**MC**^**’**) akin to the early-eluting aminopentol isomer (**I’**), which was found, based on mass spectra, in a culture of *Methylovulum*-like strain M200 [46].

### Methanotroph BHP signatures

Four previously untested methanotrophs isolated from marine or saline, alkaline lacustrine environments, belonging to the three genera *Methylomarinum*, *Methylomarinovum*, and *Methylomicrobium*, were analysed for their BHP composition. An additional species *Methylomicrobium alcaliphilum*, the partial BHP composition of which was recently reported in [53], is also shown here in full for comparison with *Methylomicrobium kenyense*. All of the methanotroph cultures investigated only synthesised BHPs with a nitrogen atom at C-35 position (nitrogen-containing BHPs herein). The relative abundances of BHPs are indicated as the percentage of total BHPs in acetylated extracts, and are presented in Fig 2. The low starting mass of some of the dry cell material led to uncertainty in the calculations of absolute concentrations, which are therefore not reported.

#### BHP inventory of *Methylomarinum vadi* IT-4

The most abundant BHPs in *Methylomarinum vadi* IT-4 were aminotriol (**III**), 22.3% and MC-triol (**III**^**MC**^), 22.7% (Fig 2A). Aminopentol (**I**), MC-pentol (**I**^**MC**^), aminotetrol (**II**), and MC-aminotetrol (**II**^**MC**^) made up 15.4%, 14.0%, 11.1%, and 11.8% of total BHPs, respectively, with lower levels of the aminopentol isomers (**I’** and **I**^**MC**^**’**; Fig 2A). No C-3 methylated or unsaturated equivalents of aminotriol, aminotetrol and aminopentol were found in *Methylomarinum vadi* IT-4.

#### BHP inventory of *Methylomarinovum* spp

The most abundant BHP in *Methylomarinovum caldicuralii* IT-9 was aminopentol (**I**), 37.4% (Fig 2B). However, *Methylomarinovum* sp. IN45 had only 5.1% aminopentol (Fig 2C). *M*. *caldicuralii* IT-9 had 30.8% MC-pentol, 14.4% aminotetrol, and 13.3% MC-tetrol, and lower abundances of aminopentol isomer, MC-pentol isomer, aminotriol, and MC-triol (Fig 2B). The most abundant BHP in *Methylomarinovum* sp. IN45 was MC-tetrol (**II**^**MC**^), making up 51.1%. *Methylomarinovum* sp. IN45 also contained 12.7% aminotetrol, 15.6% MC-pentol, 12.1% MC-triol and lower levels of aminotriol, and the aminopentol isomers (Fig 2C). No C-3 methylated nor unsaturated equivalents of aminotriol, aminotetrol and aminopentol were present in either strains.

#### BHP inventory of *Methylomicrobium* spp

The *Methylomicrobium alcaliphilum* and *Methylomicrobium kenyense* cultures did not contain aminopentol (**I**) above detection limit (Fig 2D and 2E) although *M*. *kenyense* was found to contain minor abundance of 3-Me-aminopentol (**I**^**3Me**^; 1.0%; Fig 2E). The most abundant BHP in both *Methylomicrobium* cultures was aminotriol (**III**), making up ca. 65% of all BHPs in both species. The second most abundant BHP was 3-Me-aminotriol (**III**^**3Me**^) at 31.5% in *M*. *alcaliphilum* and slightly less in *M*. *kenyense* (23.9%). Both species also contained lower levels of aminotetrol (**II**) and 3-Me-aminotetrol (**II**^**3Me**^). *M*. *kenyense* also contained unsaturated compounds (**ΔIII** and **ΔIII**^**3Me**^; Fig 2E). The only MC compound identified in either *Methylomicrobium* sp. was MC-triol and then only at low levels (<3%).

### Marine sediment and carbonate BHP signatures

Eight marine settings were studied for their BHP signatures (Table 2). Six of these were known to be influenced by CH_4_ (i.e., HHMV, BSCC, AMV, NZS, GoM cold seeps, GD) and two were used as comparison background marine levels (i.e., GoM sediments, PM).

**Table 2:** Concentrations (μg/g sediment) of amino-BHPs in marine sediment samples.

		Amino-BHPs													
		I	ΔI	I^MC^	I^3Me^	II	II^MC^	II^3Me^	III	ΔIII	III^MC^	III^3Me^	ΔIII^MC^	ΔIII^3Me^	Total nitrogen- containing BHPs (ug/g sediment)
	Base Peak^a^	830	828	846	844	772	788	786	714	712	730	728	728	726	
**Håkon Mosby Mud Volcano (HMMV)**	** **													** **	** **
new mud flow 0–1 cm	PS74/2 169–1 PUC3 229	bdl	bdl	bdl	bdl	**0.02**	**0.01**	bdl	**0.17**	**0.02**	**0.14**	bdl	**0.01**	bdl	**0.38**
aged flow 0–1 cm	ARK XXIV-2 PS74 172–1 237	bdl	bdl	bdl	bdl	**0.01**	**0.01**	bdl	**0.29**	**0.05**	**0.81**	bdl	**0.13**	bdl	**1.3**
center 0–1 cm	MSM16/2 847–1 MUC 53	bdl	bdl	bdl	bdl	**0.01**	**0.01**	bdl	**0.63**	**0.09**	**1.26**	bdl	**0.19**	bdl	**2.19**
center 10–12 cm	MSM16/2 HMMV 63	bdl	bdl	bdl	bdl	**0.01**	**0.02**	bdl	**0.17**	**0.02**	**0.17**	bdl	**0.02**	bdl	**0.41**
new mud flow 0–1 cm	MSM16/2 838–1 MUC 33	bdl	bdl	bdl	bdl	**0.02**	**0.01**	bdl	**1.07**	**0.09**	**0.82**	bdl	**0.06**	bdl	**2.07**
newer mud flow 0–1 cm	MSM16/2 855–1 MUC 73	bdl	bdl	bdl	bdl	**0.01**	**0.01**	bdl	**1.1**	**0.13**	**1.24**	bdl	**0.15**	bdl	**2.64**
newer mud flow 10–12 cm	MSM16/2 HMMV 83	bdl	bdl	bdl	bdl	**0.02**	**0.01**	bdl	**0.17**	**0.02**	**0.11**	bdl	**0.01**	bdl	**0.33**
aged flow 0–1 cm	MSM16/2 823–1 MUC 19	bdl	bdl	bdl	bdl	**0.02**	**0.02**	bdl	**0.56**	**0.05**	**0.88**	bdl	**0.07**	bdl	**1.61**
aged flow 10–12 cm	MSM16/2 HMMV 28	bdl	bdl	bdl	bdl	**0.01**	**0.01**	bdl	**0.15**	**0.01**	**0.11**	bdl	**0.01**	bdl	**0.29**
**Barents Sea Carbonate Crusts (BSCC) depth profile**															
0–2 cmbsf		bdl	bdl	bdl	bdl	**1.36**	bdl	bdl	**4.45**	**0.12**	bdl	bdl	bdl	bdl	**5.93**
2–4 cmbsf		bdl	bdl	bdl	bdl	**0.21**	bdl	bdl	**0.93**	**0.06**	bdl	bdl	bdl	bdl	**1.2**
4–6 cmbsf		bdl	bdl	bdl	bdl	**0.04**	bdl	bdl	**0.27**	**0.03**	**0.02**	bdl	bdl	bdl	**0.36**
6–8 cmbsf		bdl	bdl	bdl	bdl	**0.03**	bdl	bdl	**0.19**	**0.02**	**0.02**	bdl	bdl	bdl	**0.26**
8–10 cmbsf		bdl	bdl	bdl	bdl	bdl	bdl	bdl	**0.07**	bdl	**0.02**	bdl	bdl	bdl	**0.09**
10–13 cmbsf		bdl	bdl	bdl	bdl	bdl	bdl	bdl	**0.03**	bdl	**0.01**	bdl	bdl	bdl	**0.04**
13–16 cmbsf		bdl	bdl	bdl	bdl	bdl	bdl	bdl	**0.08**	bdl	bdl	bdl	bdl	bdl	**0.08**
16–19 cmbsf		bdl	bdl	bdl	bdl	bdl	bdl	bdl	**0.11**	bdl	bdl	bdl	bdl	bdl	**0.11**
**Amon Mud Volcano (AMV)**	** **														
central dome 0–1 cm	M70/2a 760 PUC33 24	bdl	bdl	bdl	bdl	**0.02**	**0.07**	bdl	**0.42**	**0.13**	**2.67**	bdl	**0.96**	bdl	**4.27**
sulfur band 0–1 cm	M70/2a 765 PUC49+50 71	**0.18**	bdl	**0.12**	bdl	**0.38**	**0.27**	bdl	**1.24**	**0.81**	**1.33**	bdl	**1.02**	bdl	**5.35**
sulfur band 10–12 cm	M70/2 81	**0.36**	**0.04**	**0.11**	bdl	**0.3**	**0.11**	bdl	**1.07**	**1.09**	**0.48**	bdl	**0.5**	bdl	**4.06**
sulfur band 0–1 cm	M70/2a 790 PUC68 172	**0.36**	bdl	**0.11**	bdl	**1.49**	**0.49**	bdl	**3.82**	**1.29**	**1.49**	bdl	**0.61**	bdl	**9.67**
sulfur band, white mat	MSM13/3 947–1 PUC31 73D	**0.09**	bdl	**0.11**	bdl	**0.31**	**0.24**	bdl	**0.64**	**0.36**	**0.71**	bdl	**0.44**	bdl	**2.9**
sulfur band 0–1 cm	MSM13/3 968–1 PUC15 122	**0.08**	bdl	**0.06**	bdl	**0.75**	**0.49**	bdl	**0.73**	**0.46**	**0.77**	bdl	**0.28**	bdl	**3.62**
bacterial mats 0–1 cm	MSM13/3 929–1 PUC22 45D	**0.05**	bdl	**0.01**	bdl	**1.13**	**0.17**	bdl	**1.11**	**0.43**	**0.5**	bdl	**0.16**	bdl	**3.56**
bacterial mats 10–12 cm	MSM13/3 50D	bdl	bdl	bdl	bdl	**0.01**	bdl	bdl	**0.03**	bdl	**0.02**	bdl	bdl	bdl	**0.07**
gassy centre, 10–12 cm	MSM13/3 61D	bdl	bdl	bdl	bdl	**0.01**	bdl	bdl	**0.04**	**0.01**	**0.02**	bdl	**0.01**	bdl	**0.08**
**New Zealand Seeps (NZS)**															
0–2 cmbsf	Frenulata 45	bdl	bdl	bdl	bdl	**0.09**	**0.06**	bdl	**0.21**	**0.03**	**0.29**	bdl	**0.05**	bdl	**0.73**
10–12 cmbsf	Frenulata 45	bdl	bdl	bdl	bdl	**0.13**	**0.03**	bdl	**0.26**	**0.04**	**0.15**	bdl	**0.02**	bdl	**0.63**
0–2 cmbsf	Ampharetidae 309	**0.06**	bdl	bdl	bdl	**1.11**	**0.17**	bdl	**1.32**	**1.17**	**2.59**	bdl	**0.26**	bdl	**6.67**
10–12 cmbsf	Ampharetidae 309	bdl	bdl	bdl	bdl	**0.01**	**0.02**	bdl	**0.08**	**0.02**	**0.13**	bdl	**0.02**	bdl	**0.28**
0–2 cmbsf	Sulfur-oxidising bacteria 315	**0.02**	bdl	bdl	bdl	**0.66**	**0.32**	bdl	**2.24**	**0.23**	**2.13**	bdl	**0.21**	bdl	**5.8**
10–12 cmbsf	Sulfur-oxidising bacteria 315	bdl	bdl	bdl	bdl	**0.03**	bdl	bdl	**0.14**	**0.02**	**0.1**	bdl	bdl	bdl	**0.28**
**Golfo Dulce (GD) Surface Sediment**															
10 m water depth	SG 1	bdl	bdl	bdl	bdl	bdl	bdl	bdl	**0.01**	bdl	bdl	bdl	bdl	bdl	**0.01**
24 m water depth	SG 2	**0.16**	bdl	bdl	bdl	**0.03**	bdl	bdl	**0.36**	bdl	**0.02**	bdl	bdl	bdl	**0.57**
77 m water depth	SG 4	**0.02**	bdl	bdl	bdl	**0.01**	bdl	bdl	**0.15**	**0.03**	**0.01**	bdl	bdl	bdl	**0.23**
90 m water depth	SG 5	**0.04**	bdl	bdl	bdl	**0.02**	bdl	bdl	**0.17**	**0.05**	**0.01**	bdl	bdl	bdl	**0.29**
110 m water depth	SG 6	bdl	bdl	bdl	bdl	**0.01**	bdl	bdl	**0.08**	**0.07**	**0.02**	bdl	bdl	bdl	**0.18**
120 m water depth	SG 7	**0.04**	bdl	bdl	bdl	**0.02**	bdl	bdl	**0.36**	**0.2**	**0.02**	bdl	bdl	bdl	**0.63**
140 m water depth	SG 8	**0.01**	bdl	bdl	bdl	**<0.01**	bdl	bdl	**0.11**	**0.08**	**0.01**	bdl	bdl	bdl	**0.21**
**Gulf of Mexico (GoM) Cold Seeps**	** **														
GC 234 CNSTS 4434		bdl	bdl	bdl	bdl	**0.01**	bdl	bdl	**0.09**	bdl	bdl	bdl	bdl	bdl	**0.1**
234 4436		bdl	bdl	bdl	bdl	**<0.01**	bdl	bdl	**0.04**	bdl	bdl	bdl	bdl	bdl	**0.04**
GC 234 tube worm roots 4435		bdl	bdl	bdl	bdl	**<0.01**	bdl	bdl	**0.02**	bdl	bdl	bdl	bdl	bdl	**0.02**
GC 232		bdl	bdl	bdl	bdl	**0.01**	bdl	bdl	**0.01**	bdl	bdl	bdl	bdl	bdl	**0.02**
GC 185		bdl	bdl	bdl	bdl	**0.76**	bdl	bdl	**0.2**	bdl	bdl	bdl	bdl	bdl	**0.95**
**GoM Sediments**															
West Gulf	WG2-099/6	bdl	bdl	bdl	bdl	**<0.01**	bdl	bdl	**0.01**	bdl	bdl	bdl	bdl	bdl	**0.01**
26°50'N, 92°40'W	WG2-099/9	bdl	bdl	bdl	bdl	bdl	bdl	bdl	**<0.01**	bdl	**<0.01**	bdl	bdl	bdl	**0.01**
Eastern Gulf	88-C-1 DCS 128/17	bdl	bdl	bdl	bdl	bdl	bdl	bdl	**0.01**	bdl	**0.01**	bdl	bdl	bdl	**0.02**
27°30'N, 87°20'W	DCS 128/13	bdl	bdl	bdl	bdl	bdl	bdl	bdl	**<0.01**	bdl	**<0.01**	bdl	bdl	bdl	**0.01**
	DCS 128/21	bdl	bdl	bdl	bdl	bdl	bdl	bdl	**<0.01**	bdl	**0.01**	bdl	bdl	bdl	**0.01**
Central Gulf	CGD-136/17	bdl	bdl	bdl	bdl	bdl	bdl	bdl	**0.01**	bdl	**0.01**	bdl	bdl	bdl	**0.01**
28°20'N, 89°38'W	CGD-136/13	bdl	bdl	bdl	bdl	bdl	bdl	bdl	**<0.01**	bdl	**0.01**	bdl	bdl	bdl	**0.01**
**Peru Sediment**															
10–15 cmbsf		bdl	bdl	bdl	bdl	bdl	bdl	bdl	**0.09**	bdl	bdl	bdl	bdl	bdl	**0.09**
20–25 cmbsf		bdl	bdl	bdl	bdl	bdl	bdl	bdl	**0.02**	bdl	bdl	bdl	bdl	bdl	**0.02**
40–45 cmbsf		bdl	bdl	bdl	bdl	bdl	bdl	bdl	**0.09**	bdl	bdl	bdl	bdl	bdl	**0.09**

^a^base peak = [M + H–CH3COOH]+

bdl–below detection limit

#### Håkon Mosby Mud Volcano (HMMV)

None of the HMMV sediment samples contained aminopentol (Table 2). The most abundant amino- and MC-BHPs in HMMV samples was either aminotriol (**III**) or MC-triol (**III**^**CME**^), making up 22–52% and 32–62% of total nitrogen-containing BHPs, respectively. Unsaturated aminotriol, unsaturated MC-triol, aminotetrol, and MC-tetrol all made up <10% of total nitrogen-containing BHPs. Some HMMV samples contained relatively high concentration of anhydrobacteriohopanetetrol (anhydro-BHT). Minor abundances of BHT and BHT isomer, BHT-cyclitol ether (BHT-CE), and BHT-glucosamine (BHT-G) were detected in some samples (S1 Table).

#### Barents Sea carbonate crust (BSCC)

The 19 cm BSCC core contained a majority of aminotriol (**III**; 72–100% of nitrogen-containing BHPs; Table 2). MC-triol (**III**^**MC**^; 0–23%) peaked between 4 and 13 cm. Aminotetrol (**II**; 0–23%) was detected in the upper 8 cm. Minor contribution of unsaturated aminotriol (**ΔIII**; 0–7%) was detected in the upper sediments. Other BHPs detected included BHT, and low concentrations of anhydroBHT, BHT isomer, 2-methyl-BHT (2-Me-BHT), adenosylhopane, BHT-CE, and BHT-G (S1 Table).

#### Amon Mud Volcano (AMV)

The most abundant nitrogen-containing BHPs in sediments from the Amon mud volcano were aminotriol (**III**; 9.8–48.9%) and MC-triol (**III**^**MC**^;11.8–62.5%) (Table 2). Other nitrogen-containing BHPs in these sediments were aminotetrol (**II**; 0.5–31.8%), unsaturated aminotriol (**ΔIII**; 3.0–27.0%) and unsaturated MC-triol (**ΔIII**^**MC**^; 0–22.5%). Minor abundances of aminopentol (**I**; 0–8.9%), and MC-pentol (**I**^**MC**^; 0–3.8%) were found in some AMV sediments. Other BHPs in the AMV sediments were BHT, anhydroBHT, 2-Me-BHT, 3-Me-BHT, BHT-CE, BHT-G, BHT isomer, and adenosylhopane (S1 Table).

#### New Zealand Seeps (NZS)

The most abundant nitrogen-containing BHP in the sediments from NZS were aminotriol (**III**; 28.5–72.7%), MC-triol (**III**^**MC**^; 13.2–46.4%) and aminotetrol (**II**; 5.1–20.6%) (Table 2). Unsaturated aminotriol (**ΔIII**), unsaturated MC-triol (**ΔIII**^**MC**^), and MC-tetrol (**II**^**MC**^) all made up <10%. Two sediment samples contained aminopentol, albeit in low abundance (<1% of nitrogen-containing BHPs). Both of these aminopentol positive samples also contained soil marker BHPs [77,82]. Other BHPs found in NZS samples were BHT, anhydroBHT, 2-methyl-BHT, 3-methyl-BHT, BHT isomer, adenosylhopane, and BHT-CE (S1 Table).

#### Gulfo Dulce (GD)

The most abundant nitrogen-containing BHP in GD surface sediments was aminotriol (**III**; 44.3–100%; Table 2). Unsaturated aminotriol (**ΔIII**) made up 0–41.3%. MC-triol (**III**^**MC**^; 0–11.4%) and aminotetrol (**II**; 0–6.0%) were found in most samples. Aminopentol was found in all but two surface samples, one of which was the shallowest site. Aminopentol abundance decreased with increasing water depth with the highest abundance at 24 m water depth (27.6% of nitrogen-containing BHPs). BHT, anhydroBHT, BHT isomer, and 2-Me-BHT were also found in GD (S1 Table).

#### Gulf of Mexico (GoM) cold seeps

GoM cold seeps only contained aminotriol (**III**; 20.5–100%) and aminotetrol (**II**; 0–79.5%) nitrogen-containing BHPs (Table 2). Other BHPs included BHT, soil marker BHPs, 2-Me-BHT, BHT isomer, and BHT-CE (S1 Table).

#### GoM sediments

GoM sediments from near the outflow of Mississippi River Delta showed an abundance of aminotriol (**III**; 26.6–80.7% of total nitrogen-containing BHPs), MC-triol (**III**^**MC**^; 0–73.4%) (Table 2). One sample from the Western Basin had 19.3% aminotetrol (**II**) relative to total BHPs; however, the concentration of aminotetrol was low (<0.01 μg/g sediment). Other BHPs included BHT, BHT isomer, anhydroBHT, 2-methyl-BHT, BHT-CE, and soil marker BHPs (S1 Table).

#### Peru Margin (PM)

The only nitrogen-containing BHP detected in sediments from the PM was aminotriol (**III**). Other BHPs found at this site included BHT, BHT isomer, anhydroBHT, 2-Me-BHT, adenosylhopane, and BHT-CE (S1 Table).

## Discussion

### BHP distributions in aerobic methanotrophs

#### Previously reported BHP distributions in AMO bacteria

**Fig 3:**
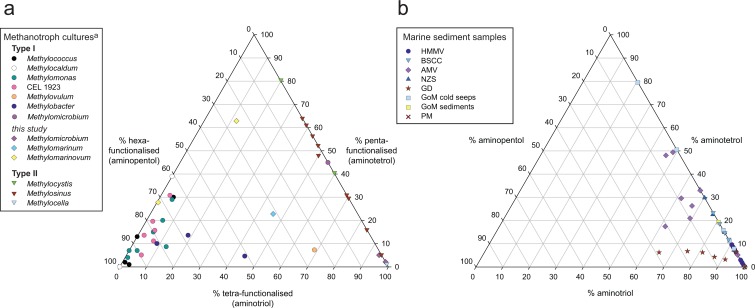
Relative composition (%) of ‘regular’ amino-BHPs. Distributions of aminotriol, aminotetrol and aminopentol, including their C-3 methylated homologues, where present, are shown for (a) methanotroph cultures including literature data (circles) and data from new cultures of *Methylomicrobium*, *Methylomarinum*, and *Methylomarinovum* (diamonds; this study) and (b) sediments and microbial mats from methane-rich marine settings (HMMV = Håkon Mosby mud volcano; BSCC = Barents Sea carbonate crust; AMV = Amon mud volcano; NZ = New Zealand; GD Golfo Dulce; GoM = Gulf of Mexico; PM = Peru Margin). Methanotroph literature data from [36,42,46,52,83]. ^a^Literature data from [36,42,83] was based on GC-MS hopanol quantifications after periodic acid treatment. Therefore, it is not possible to distinguish between amino- and non-amino-BHPs with the same number of functional groups. This is especially significant for the Type II aerobic methanotrophic bacteria that are known to synthesise bacteriohopanetetrol. For this reason, the axes of plot (a) are shown as functionality of the BHP-side chain.

Traditionally, Type I and Type II AMO bacteria had been distinguished by their different BHP signatures (e.g. [52]; see also review in [14]). Prior to the investigation of BHPs in the *Methylovulum*-like strain M200 [46], most screened Type I methanotrophs synthesised a high percentage of aminopentol (**I**) and lower contributions from aminotetrol (**II**) and in some cases aminotriol (**III**), and clustered in the left-hand corner of the amino-BHP ternary plot (Fig 3A). In contrast, Type II methanotrophs did not contain aminopentol, had varying contributions from **II** and **III**, and clustered along the right-hand axis of Fig 3A. The high relative abundance of **III** observed in *Methylovulum*-like strain M200 was, therefore, originally seen as an outlier [46]. Similarly, [52] showed that a culture of *Methylomicrobium album* did not contain aminopentol. At the time this was presumed to be a contaminated culture, however, [53] also did not report **I** synthesis in cultures of *Methylomicrobium alcaliphilum*. All of the recently analysed *Methylobacter* spp. [28] join the more typical Type I methanotrophs in the left-hand corner of the plot, however, *Methylobacter* sp. BB5.1 increased the spread of the cluster with almost 40% **III** content.

#### Amino-BHP distributions in previously untested Type I AMO bacterial cultures

It was assumed that the screened species of AMO Type I bacteria investigated in this study would display similar BHP distributions as those of previously reported Type I bacteria. All three bacterial genera screened do indeed only contain amino-BHPs (Fig 2). However, the relative distribution of specific nitrogen-containing BHPs varies between genera, as well as between species belonging to the same genus. To allow for a more accurate comparison with data from the literature (Fig 3A, circles), only aminopentol, aminotetrol, aminotriol, and their methylated equivalents were considered when producing the ternary plot of ‘regular’ nitrogen-containing BHPs of the novel Type I cultures (Fig 3A, diamonds).

Aminopentol is the most abundant BHP in the novel species *Methylomarinovum cadicuralii* IT-9, which is in agreement with literature BHP compositions of most other Type I methanotrophs (Fig 3A, circles), e.g., *Methylococcus capsulatus* [44,52], *Methylomonas* sp. [42,46,52], psychrotolerant isolate CEL 1923 [42], and *Methylocaldum tepidum* [22]. However, a species in the same genus (*Methylomarinovum* sp. IN45) has a much lower abundance of aminopentol (5.0% of total amino-BHPs; Fig 2C). *Methylomarinum vadi* IT-4 shows relatively high proportions of aminopentol, but it is not the most abundant BHP (Fig 2A). Moreover, in our screening of two species of *Methylomicrobium* spp., aminopentol was not detected, similar to reported cultures of *Methylomicrobium album* and *Methylomicrobium alcaliphilum* [52,53]. Our results seem to confirm the near-absence of aminopentol in all screened *Methylomicrobium* spp., which are the first Type I methanotrophs apparently unable to synthesise aminopentol. However, changes in BHP composition can occur at different growth stages and under different conditions (e.g. [27,83,84]), so further studies would be required to fully confirm this. It appears that the BHP distributions of *Methylomicrobium* and *Methylovulum*, which do not synthesise high amounts of aminopentol, should also no longer be considered outliers given the low levels of aminopentol in *M*. *vadi* IT-4 and *Methylomarinovum* spp. (Fig 2). This suggests a greater variance in the BHPs of Type I methanotrophs than previously thought. Furthermore, as *Methylomicrobium* has been isolated from a diverse range of marine environments [85–87], the absence of aminopentol in this genus might greatly affect its application as a marine aerobic methanotrophy biomarker. This, however, does not invalidate the use of aminopentol as a biomarker for methanotrophy.

There is also significant variation in the relative abundances of the other nitrogen-containing BHPs in Type I methanotrophs. A suite of novel BHPs identified as methylcarbamate (MC) BHPs are detected in all three genera screened in this study (Fig 2). These have not been reported in previous studies. Therefore, data available at Newcastle University from the analyses of *Methylococcus capsulatus* (Talbot et al., unpublished), *Methylovulum*-like strain M200, *Methylomonas methanica*, *Methylomonas*-like strain M5 [46], and *Methylobacter* spp. [28] were re-examined. **III**^**MC**^ was identified retrospectively in *Methylomonas methanica*, and all species of *Methylobacter*. **III**^**MC**^ constituted up 9.8% of total amino-BHPs in *Methylobacter* sp. BB 5.1. **II**^**MC**^ was present in low abundance (<0.2%) in two of the three species of *Methylobacter (Methylobacter* sp. BBA 5.1 and *Methylobacter* sp. BB 5.1), but absent in *Methylobacter marinus* A 45. **I**^**MC**^ was absent in *Methylococcus capsulatus* and *Methylovulum*-like strain M200, but was present in all species of *Methylobacter*. **I**^**MC**^ was also present in *Methylomonas methanica* but absent in a different species of this genus (i.e., *Methylomonas*-like strain M5; [46]). These results indicate that MC-BHPs are not universally present when their regular homologues are detected, and may be species specific and/or dependent on variations in growth conditions such as pH.

The two species of *Methylomarinovum* display significant variations in their BHP compositions (Fig 2B and 2C). *Methylomarinovum* sp. IN45 had a relatively low level of aminopentol. The most abundant BHP in *Methylomarinovum* sp. IN45 is **II**^**MC**^. Although in lower abundances, **III**^**MC**^ **and I**^**MC**^ are also higher in comparison to their ‘regular’ homologues in this species compared to *M*. *caldicuralii* IT-9 (Fig 2B and 2C). In contrast, the most abundant BHP in *Methylomarinovum caldicuralii* IT-9 is aminopentol, followed by almost equal amounts of **I**^**MC**^. The different relative BHP distributions between the *Methylomarinovum* spp. highlight that there can be significant variations within a genus. *Methylomarinovum* sp. IN45 was isolated from a deep-sea hydrothermal field and perhaps the high levels of methylcarbamate components observed are the result of a physiological adaptation to higher pressure in this environment. This may explain why the relative abundances of components in *Methylomarinovum caldicuralii* IT-9, the same genus but isolated from a shallow submarine hydrothermal environment, are quite different. Perhaps the complex functionality of the terminal group of the methylcarbamate components is more effective at stabilising the cell membrane and decreasing fluidity under these conditions.

3-methylaminotriol (**III**^**3Me**^) was observed in both *Methylomicrobium* spp. (23.9–31.5% of total BHPs; Fig 2D and 2E) in agreement with a recent report in [53]. This compound was accompanied by low levels of 3-methylaminotetrol (**II**^**3Me**^) in both species and trace amounts of 3-methylaminopentol in *M*. *kenyense* (**I**^**3Me**^). The absence of C-3 methylated structures in the previously investigated *Methylomicrobium album s*train BG8 [52] may appear inconsistent with the organisms investigated here; however, genomic investigations have revealed that *M*. *album* is separated from halo(alkali)philic representatives of the *Methylomicrobium* genus such as *M*. *alcaliphium* and *M*. *kenyense* [58], and perhaps specific environmental conditions influence the BHP composition of *Methylomicrobium* spp. as they seemingly do within the *Methylomarinovum* genus.

**Fig 4:**
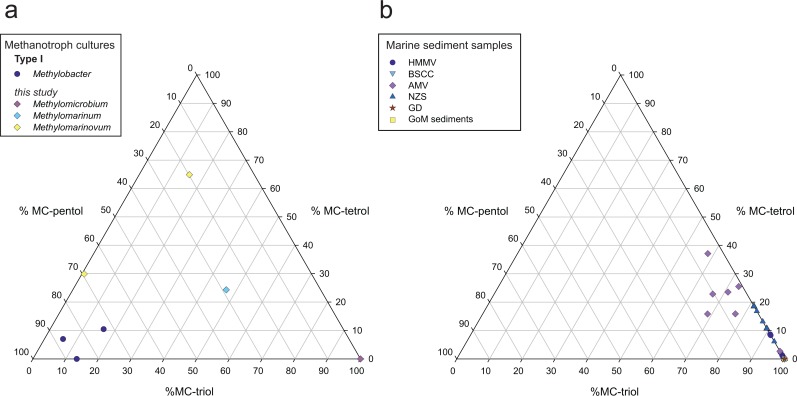
Relative composition (%) of methylcarbamate(MC)- BHPs. Distributions of MC-triol, MC-tetrol and MC-pentol are shown for (a) methanotroph Type I cultures, *Methylobacter*, *Methylomicrobium*, *Methylomarinum*, and *Methylomarinovum*, and (b) marine sediments (HMMV = Håkon Mosby mud volcano; BSCC = Barents Sea carbonate crust; AMV = Amon mud volcano; NZ = New Zealand; GD Golfo Dulce; GoM = Gulf of Mexico). *Methylobacter* data from [28].

No C-3 methylated equivalents of aminotriol (**III**^**3Me**^), aminotetrol (**II**^**3Me**^), nor aminopentol (**I**^**3Me**^) were present in *Methylomarinum vadi* IT-4 or the *Methylomarinovum* spp., adding to examples of Type I methanotroph species that contain amino-BHPs, but not their C-3 methylated equivalents (e.g., see review in [14]). The most abundant BHPs in *Methylomarinum vadi* IT-4 were aminotriol (**III**) and MC-triol (**III**^**MC**^), which were present in equal amounts (Fig 2A). Similar amounts of **II** and **II**^**MC**^, and **I** and **I**^**MC**^ were also observed in this culture. A high proportion of **III** is unusual for a Type I methanotroph, but has been observed before in the *Methylovulum*-like strain M200. (Fig 4A; [46]). The new data reiterate that aminopentol is not always the most abundant BHP in Type I methanotrophs, nor necessarily the most appropriate biomarker for AMO.

### BHPs in marine environments

#### Lack of BHP diversity in marine environments

The screened marine sediments and authigenic carbonates do not show large diversity in their BHP signatures (S1 Table). The limited BHP distributions are also comparable to other reported marine sediment BHP signatures, all dominated by BHT and BHT isomer, from a number of locations including the Black Sea [88], the Benguela upwelling system [89], and the Arabian Sea [90]. More recently a similar pattern was also seen in water column samples from the California Current system, where the wide diversity observed in the gene responsible for hopane cyclisation (squalene-hopene cyclase) was not reflected by distinct BHP fingerprints related to this potential range of source-organisms [91]. However, genetic information is quickly lost, and we must strive to find lipid biomarkers to trace particular metabolisms in the geological record.

Non-nitrogen-containing BHP concentrations in the screened sediments do not show remarkable signatures (S1 Table). BHT and anhydro-BHT, thought to be a degradation product of BHT and other composite BHPs such as BHT cyclitol ether [92], were found at all sites. The presence of soil marker BHPs at some sites, particularly NZS, indicates that these sediments could be influenced by terrestrial input of organic matter (e.g., [77,82,93]). However, as adenosylhopane is an intermediate in the biosynthesis of all other side-chain extended BHPs [94], other sources cannot be entirely excluded. BHT isomer, a biomarker for anaerobic ammonium oxidation [70], was found in high concentrations in GD (previously reported in [70]), as well as in the PM sediments, which underlie the Peruvian OMZ, where anammox is known to be an important process [95], and where BHT isomer has previously been reported from the water column within the OMZ [90]. The most abundant of the three regular amino-BHPs in the CH_4_-influenced marine sediments was aminotriol, which is not source-specific (e.g., [48]).

#### Aminopentol in marine sediments

Although aminopentol was found in significant abundance in some of the reported and screened Type I methanotroph cultures (Fig 2), it was not found to be abundant in most of the CH_4_-influenced marine sites in this study (Table 2). In fact, it was only detected in AMV, GD surface sediments, and two NZS samples (Fig 3B; Table 2). The discrepancy between the distinct amino-BHP signatures of isolated Type I AMO bacteria and signatures of CH_4_-influenced marine sites is highlighted in the ternary plots of the relative composition of aminopentol, aminotetrol, and aminotriol (Fig 3A cf. Fig 3B). These differences could be due to the particular methanotrophic bacterial community responsible for methanotrophy in the CH_4_-influenced marine sediments. Ruff et al. [96] found that diversity in the global CH_4_ seep microbiome was controlled by environmental factors such as temperature and electron acceptor availability. Considering their findings, it is possible that the environmental conditions in most marine CH_4_-influenced sediments favour specific methanotroph communities. For example *Methylomicrobium* spp., found in saline environments [85–87] and saline, highly alkaline environments [58,97], and which do not produce aminopentol in significant amounts (Fig 2), could be present. However, the absence of C-3 methylated compounds is confounding for a *Methylomicrobium* source, pointing towards other methanotrophs that do not synthesise aminopentol. Yan et al. [98] found that 85% of the operational taxonomic units (OTUs) from the same sites as our GoM cold seeps did not group with known sequences of a subunit of particulate methane monooxygenase (*pmoA*). This would suggest the presence of novel methanotrophic species in GoM. In the same way, significant *pmoA* diversity has been observed in sediments from the North American margin [99], a shallow CH_4_ seep [100], a marine estuary [101], and hydrocarbon seeps [102]. *pmoA* OTUs from the NZS sediments grouped with methanotrophic endosymbionts [68], including *Bathymodiolus* spp., which have been shown to contain neither aminopentol nor methylated BHPs [103]. Nevertheless, the absence of methylated amino-BHPs in the screened marine sediments (Table 2) may suggest *Methylomicrobium album*, or a related species that also does not synthesise methylated amino-BHPs, being the dominant methanotroph in CH_4_-influenced marine environments.

These are not the first reports of marine CH_4_-influenced environments not containing aminopentol (Table 1). For example, using methods targeting the functional gene *pmoA*, which is produced by most methanotrophs, Type I methanotrophs were detected in all three units of Ace Lake sediments. However, aminopentol was only detected in sediments deposited under freshwater conditions (unit III) despite the fact that the modern meromictic water column, containing relict seawater left behind after the sea level fell around 9000 years ago, hosts the Type I methanotroph *Methylosphaera hansonii* [104]. No aminopentol was detected in the methanotrophic symbionts in the gill tissue of a cold-seep mussel, despite other lipid-based evidence suggesting the presence of a Type I methanotroph [103,105]. Similarly, CH_4_ seep carbonates from Alaminos Canyon, northern Gulf of Mexico [41] and the Northern Arabian Sea [106] were found to lack aminopentol. Conversely, aminopentol was detected in the water column of the Baltic Sea with supporting evidence for the presence of Type I methanotrophs from ^13^C-depleted PLFAs [107]. Aminopentol was also detected in the water column of the Black Sea in the oxic-anoxic water transition, but not in the underlying sediment [12,88,108].

The presence of aminopentol in sediments from the AMV, located on the Nile deep-sea fan, in the Eastern Basin of the Mediterranean Sea (Table 2) may be explained by Nile River outflow carrying terrestrial wetland methanotrophy signatures into the Mediterranean, as seen in the Amazon and Congo River fans [14,109]. This would appear to indicate that aminopentol is still an excellent biomarker for terrestrial AMO. The near-absence of soil-marker BHPs in AMV (Table 2) may still point towards in-situ marine production of aminopentol. However, the relative abundance of soil-markers in terrestrial settings has recently been found to be strongly influenced by environmental factors; higher temperatures and low pH (in peatlands) can both strongly reduce the relative proportion of soil marker BHPs as a proportion of total BHPs [40,110,111]. Aminopentol was found in NZS sediments that also contained soil marker BHPs (Table 2). Therefore, aminopentol in sediments from NZS may have originated from terrestrial sources. Aminopentol in GD surface sediments may be the result of a distinct AMO community living in the specific environment prone to carbonate formation in GD. Unfortunately, samples were not properly preserved to be able to determine AMO diversity using genetic-based analyses of the *pmoA* gene in these sediments. The cumulative results of the studied marine sites do, however, indicate that an absence of aminopentol is not necessarily evidence for the absence of methanotrophs or aerobic methane oxidation.

### Alternative BHP biomarkers for AMO and implications for the marine sedimentary record of methanotrophy

#### Regular amino-BHPs

Screened Type I methanotrophs also produced varying amounts, depending on the genera, of aminotetrol (**II**) and aminotriol (**III**) (Fig 3A), both of which were found in CH_4_-influenced marine sediments (Fig 3B; Table 2). However, these two amino-BHPs are less source-specific to methanotrophic bacteria than aminopentol, and do not make ideal biomarker lipids for methanotrophy. Given that 3-Me-aminotriol (**III**^**3Me**^) made a significant contribution to the amino-BHP abundance in screened cultures of *Methylomicrobium* spp. (23.9 and 31.5% of total amino-BHPs; Fig 2D and 2E) and 9.8% in *Methylobacter* sp. BB5.1 [28], it was expected that **III**^**3Me**^ would be an important amino-BHP in CH_4_-influenced marine sediments. However, **III**^**3Me**^ was not found in any of the screened sediments (Table 2). *Methylomicrobium alcaliphilum* and *Methylomicrobium kenyense* are adapted to high alkalinity, but not necessarily to high salinity [58]. This distinct lack of **III**^**3Me**^ in marine sediment samples would seem to indicate that the *Methylomicrobium* species we investigated are not the primary source of amino-BHPs in CH_4_-influenced marine environments.

**III**^**3Me**^ has only occasionally been reported from environmental samples including some soils [82,112] and most recently in a peat core from Germany [111], but only at very low levels (Table 1). Other C-3 methylated amino-BHPs are even less common (Table 1). **I**^**3Me**^ was first reported from a neo-volcanic, eutrophic and saline lake sediment (La Piscina de Yuriria, Mexico; [45]), and subsequently from a geothermal silica sinter (Opaheke Pool hot spring, New Zealand; [113]). The pentafunctionalised **II**^**3Me**^ was also present in the Mexican lake sediment. Both of these compounds were reported in one study on the Black Sea water column [12], but were absent at another site [108]. The apparent discrepancy between the very limited occurrence of C-3 methylated BHPs (as measured using the periodic acid cleavage technique which converts polyfunctionalised BHPs into GC-amenable primary alcohols; e.g., [36,75]) and their wider occurrence in the form of 3-Me hopanes in ancient rocks and oils was first identified in [114]. These authors found 3-Me-BHPs to be abundant only in a very limited number of settings, under quite specific conditions (i.e., some alkaline lakes). The occurrence of 3-Me-hopanes in marine authigenic carbonates [31,115], which form under highly alkaline conditions are also consistent with a *Methylomicrobium* source ([58], and references therein). It was further suggested that 3-Me-BHPs and hexafunctionalized BHPs appear to have different sources (possibly, but not necessarily restricted to, only Type I methanotrophs; [114]). Culture studies (on the moderately thermophilic Type I methanotroph *Methylococcus capsulatus*) have shown that production of C-3 methylated compounds may be related to growth stage. Higher relative proportions of methylated BHPs replaced the non-methylated equivalents during stationary phase growth [83], and appear to be necessary for maintaining intra-cellular membrane structures [27]. These important physiological roles for methylated BHPs are at odds with the very sparse occurrence of these compounds in modern settings (Table 1), and clearly our understanding of the factors controlling their biosynthesis and subsequent preservation in sediments is still limited, hampering interpretation of certain BHP signatures.

#### Methylcarbamate-BHPs

Most of the marine sediments influenced by CH_4_ contained at least MC-triol, albeit at relatively low abundances (Fig 4B; Table 2). The fact that the MC-BHPs were found in all strains of methanotrophs analysed, though not all components in the suite were present in every strain, shows the biomarker potential of these BHPs for AMO (Fig 3A). MC-tetrol (**II**^**MC**^) was the most abundant component in *Methylomarinovum* sp. IN45, and MC-BHPs were found in higher abundance than the ‘regular’ 35-amino-BHP homologues, which may allow this particular hydrothermal vent species to be identified in environmental settings. Unsaturated MC-triol (**ΔIII**^**CME**^) was found in high abundance in AMV, HMMV, and NZS, but was not found in any of the methanotroph cultures. This is possibly because the BHP signatures in most CH_4_-influenced marine sediments are sourced from AMO bacteria that have no cultured relatives or at least none which have been tested for BHP production.

Given the small diversity in BHPs found in marine sediments and the need for an AMO biomarker, there appear to be few BHPs that meet the criteria of being source-specific *and* abundant. This has significant implications for the development of a proxy using aminopentol to trace AMO in marine settings. Applying MC-BHPs combined with the traditional suite of amino-BHPs (e.g. aminopentol, aminotetrol, and aminotriol) seems to be the most appropriate biomarker course for AMO.

## Conclusions

Isolated methanotrophs from previously unexamined genera and species displayed marked differences in their relative abundances of amino-bacteriohopanepolyols (BHPs). Aminopentol (**I**) was the most abundant BHP in *Methylomarinovum caldicuralii* IT-9, which fits with the typical BHP signature of known Type I methanotrophs. However, the BHP signatures of *Methylomarinovum* sp. IN45 and *Methylomarinum vadi* IT-4 both did not show aminopentol as the most abundant BHP. Moreover, neither of the *Methylomicrobium* spp. contained aminopentol and only one contained a low level of 3-methyl-aminopentol showing that not all Type I methanotrophs synthesise aminopentol, agreeing with previous environmental studies. Considering *Methylomicrobium* can be prevalent in marine environments, this has implications for the use of aminopentol as a biomarker for marine methanotrophy. A suite of components related to amino-BHPs, but with methylcarbamate (MC) terminal groups, were detected for the first time, and were present in all Type I methanotroph strains tested. Marine sediments influenced by CH_4_ did not contain significant amount of aminopentol, but did contain MC-BHPs. This study highlights the relatively low BHP diversity within marine sediments, and indicates that the combined use of MC-BHPs and amino-BHPs might be preferential to trace aerobic methane oxidation (AMO) in marine settings.

## Supporting Information

S1 File Identification of novel BHP compounds in methanotroph cultures. (DOCX)

S1 Table Concentrations of other BHPs in marine sediment samples. Concentration (μg/g sediment) of other BHPs in marine sediments samples presented in this study.
(XLSX)
