# Supplementary material for: The Bacteriohopanepolyol Inventory of Novel Aerobic Methane Oxidising Bacteria Reveals New Biomarker Signatures of Aerobic Methanotrophy in Marine Systems
Source: PLoS One. 2016 Nov 8;11(11):e0165635. doi: 10.1371/journal.pone.0165635 (PMC5100885; doi:10.1371/journal.pone.0165635)
Supplement: S1 File — (DOCX) [file pone.0165635.s001.docx]

**Bacteriohopanepolyol lipids of novel aerobic methane oxidising bacteria reveal biomarker signatures of aerobic methanotrophy in marine systems**

Darci Rush^1¶*^, Kate A. Osborne^1¶^, Daniel Birgel^2^, Andreas Kappler^3,4^, Hisako Hirayama^5^, Jörn Peckmann^2,6^, Simon W. Poulton^7^, Julia C. Nickel^8^, Kai Mangelsdorf^8^, Marina Kalyuzhnaya^9^, Frances R. Sidgwick^1^, Helen M. Talbot^1^

**SI File 1: Identification of novel BHP compounds in methanotroph cultures**

All of the novel methanotrophs investigated in this study contained a combination of the regular aminoBHPs previously reported from a wide range of methanotrophs including aminotriol, aminotetrol, aminopentol (**III**, **II** and **I** respectively, Figs 1, 2; e.g. Cvejic et al., 2000; Talbot et al., 2001; van Winden et al., 2012). The C-3 methylated homologues of each structure were also identified in the *Methylomicrobium* spp. (Fig 2 d,e). In addition to the regular compounds we also identified a series of related but unknown compounds, each with a peracetylated [M+H]^+^ ion 16 Da higher than their “regular” aminoBHP counterpart. Using HPLC-MS^n^ (e.g. Talbot et al., 2003b, 2007a) we observed these compounds as peaks in their respective mass chromatograms (indicated with “**^MC^**” in Fig 2, see below for explanation). These peaks, eluting just after the regular compounds in each pair, indicate a slightly less polar structure than the regular compound. Given the obvious importance of these peaks, particularly in the *Methylomarinum vadi* and *Methylomarinovum* spp. cultures (Fig 2a-c), we interrogated their ion-trap atmospheric pressure chemical ionisation (APCI) MS^2^ and MS^3^ spectra in order to attempts to deduce a possible structure.

*Identification of peracetylated tetrafunctionalised novel BHP related to aminotriol: 35-*methylcarbamate*-bacteriohopane-32,33,34-triol (****III^MC^****)*

Peak **III^MC^**, related to, and eluting after **III** (Fig. 2), has an even numbered base peak ion [M+H]^+^ of *m/z* 730, which indicates the presence of an odd number of N atoms in the molecule. The neutral loss of three acetylated hydroxyl groups, to give ion fragments of *m/z* 670, 610 and 550 (Fig SI1 – 1b; Table S1 – 1) is observed in the MS^2^ spectrum of this component as also observed for aminotriol (**III**) ([Fig SI1 – 1; Table SI1 – 1, cf. Talbot et al., 2003a](#_ENREF_70)). Further structural information was obtained from the MS^3^ spectrum from fragmentation of the *m/z* 670 ion from MS^2^ (Fig SI1 – 1b,c). The ion fragment *of m/z* 638 indicates neutral loss of 32 Da (CH_3_OH). Such a fragment has not been observed previously in the ion-trap APCI spectrum of any BHPs (Talbot et al., 2003a, b, 2005, 2007a, 2007b, 2008, 2016). Here we interpret this loss as indicating the presence of a methyl ether (lost as CH_3_OH; Fig SI1 – 1c). Given the apparent relationship of peaks **III^MC^** and **III** (based on relative retention times and similarity of the APCI MS^2^ spectra which suggests **III^MC^** is also a tetrafunctionalised compound), the methyl ether can only represent partial loss of the 4^th^ functional group which must also include an odd number of N atoms. This confirms that the fourth functional group present in this molecule is not a regular acetate derivative (either -OH or -NH_2_) as this mass loss has not been observed in previously investigated peracetylated BHPs. The ion *m/z* 475 indicates the neutral loss of 75 Da (after loss of all three regular acetylated hydroxyls) and indicates the full terminal group with the proposed structure -NHCOOCH_3_ (i.e. cabamylmethylester) which allows for the initial loss of 32 Da (Fig SI1 – 1c). Though not visible in the MS^2^ spectrum, neutral loss of the acetylated terminal group as NH_2_COCH_3_, in aminotriol (**III**; Fig SI1 – 1a) results in the loss of 59 Da and has been observed previously in the MS^3^ spectrum of this component ([Talbot et al., 2003a](#_ENREF_70)). The ion fragments of *m/z* 568 and 628 indicate the loss of COCH_2_ (ketene) from the two remaining acetylated hydroxyls (Fig SI1 – 1c). Finally, the hopanoid nature of the compound is confirmed by several minor ions in the MS^3^ spectrum including *m/z* 191 (loss of the A+B rings; e.g. Talbot et al., 2003a,b) and ions indicating neutral loss of the A+B rings (loss of 192 Da) with charge retention of the D+E+side chain containing fragment (m/z 478, 418, 358 and 283; Fig SI1 – 1c). Although present only at low intensity, and only in the MS^3^, this is fully consistent with previous studies of the MS^2^ and MS^3^ spectra of the regular aminoBHPs (Talbot et al., 2003a,b). Therfore **III^MC^** is tentatively named 35-methylcarbamate-bacteriohopane-32,33,34-triol (MC-triol from herein).


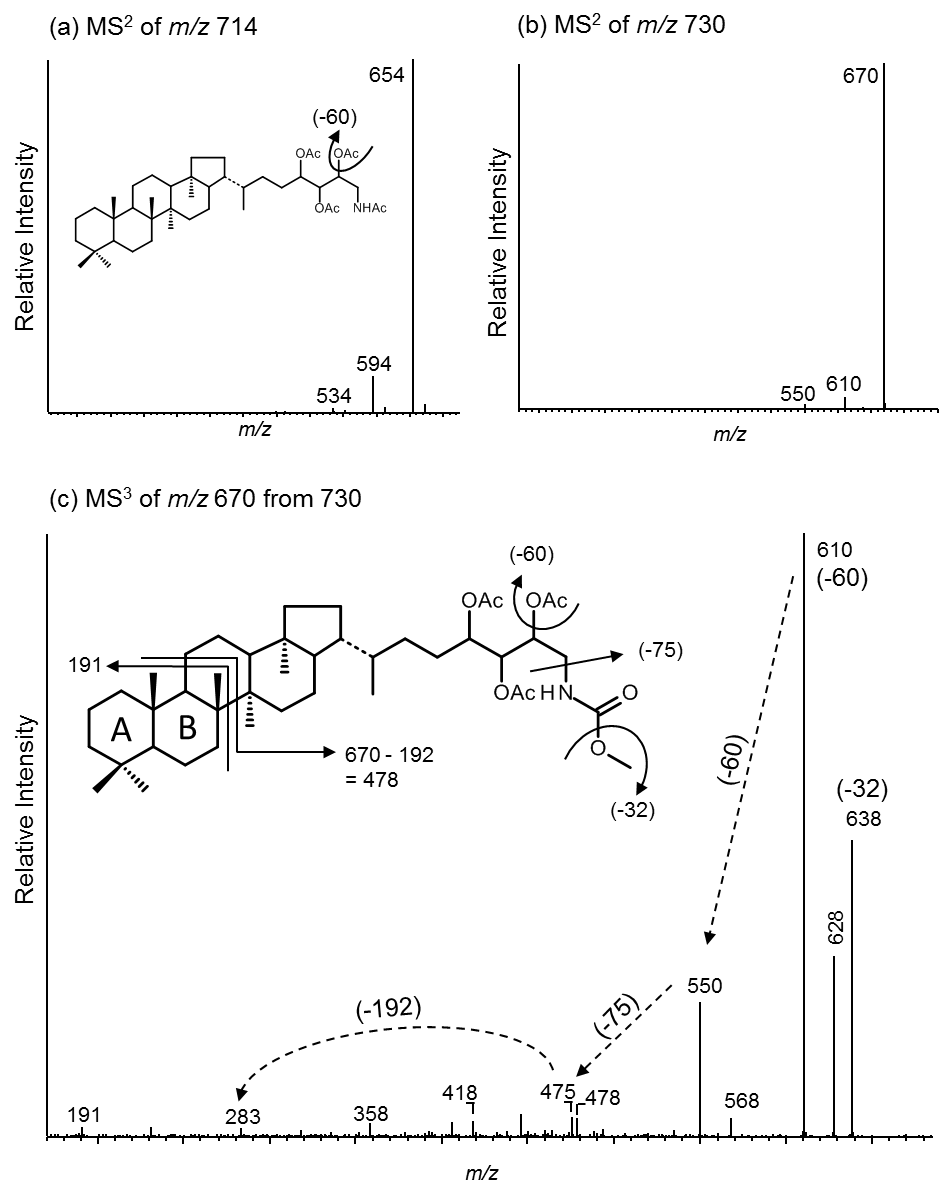


**Figure SI1 – 1.** (a) APCI MS^2^ spectrum of aminotriol (**III**); (b) APCI MS^2^ spectrum of proposed methylcarbamate-triol (**III^MC^**); (c) APCI MS^3^ spectrum of **III^MC^**. Numbers in brackets indicate neutral molecule loss (Da). Ac = COCH_3_.

*1.2 Identification of peracetylated pentafunctionalised novel BHP related to aminotetrol: 35-*methylcarbamate*-bacteriohopane-31,32,33,34-tetrol (****II^MC^****)*

Component **II^MC^**, related to, and eluting just after **II**, has a base peak ion of *m/z* 788 (Fig 2). The APCI MS^2^ spectrum indicates neutral loss of four acetylated hydroxyl groups, CH_3_COOH (ion fragments of *m/z* 728, 668, 608, 548), akin to that of aminotetrol (**II**) (Fig SI1 – 2a,b; Table S1). The MS^3^ spectrum from *m/z* 728 contains the ion fragment of *m/z* 686 indicating the loss COCH_2_ (Fig SI1 – 2). There is also a fragment of *m/z* 696 which indicates the partial loss of the terminal group, CH_3_OH, although this is significantly less intense than the equivalent ion in MC-triol (Fig SI1 – 1c), but suggests that loss of the methyl ether (as CH_3_OH) is significantly less favourable in the more highly (penta)functionalised compound that losses of the acetylated hydroxyls (m/z 608, 548). The ion fragment of *m/z* 473 indicates the neutral loss of 75 Da, equivalent to the full terminal group NH_2_COOCH_3_ (i.e. cabamylmethylester). Finally, the hopanoid nature of the compound is supported by several minor ions in the MS^3^ spectrum including *m/z* 231 which is an ion commonly reported in BHP APCI mass spectra (Talbot et al., 2003a,b, 2007a,b) and ions indicating neutral loss of the A+B rings (loss of 192 Da) with charge retention of the D+E+side chain containing fragment (m/z 536, 476 and 356; Fig SI1 – 2c). The component is therefore tentative named 35-methylcarbamate-bacteriohopane-32,33,34-tetrol (MC-tetrol herein; **II^MC^**)**.**


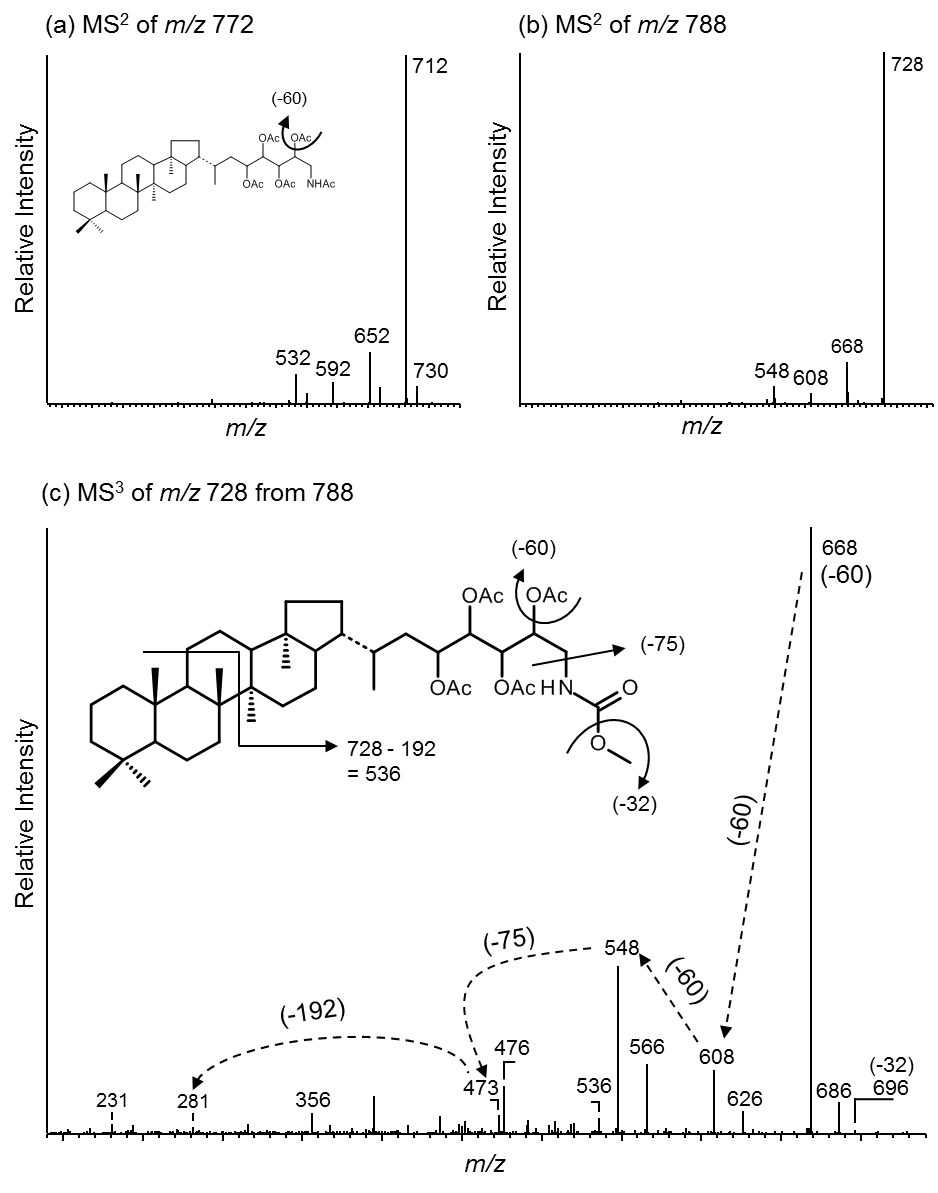


**Figure SI1 – 2.** (a) APCI MS^2^ spectrum of aminotetrol (**II**); (b) APCI MS^2^ spectrum of proposed MC-tetrol (**II^MC^**); (c) APCI MS^3^ spectrum of **II^MC^**. Numbers in brackets indicate neutral molecule loss (Da). Ac = COCH_3_.

*1.3 Identification of peracetylated pentafunctionalised novel BHP related to aminopentol:* 35-methylcarbamate-bacteriohopane-30,31,32,33,34-pentol (**I^MC^**)**.**

Component **I^MC^**, related to, and eluting after **I**, has a base peak ion of *m/z* 846. The neutral loss of five acetylated hydroxyl groups, CH_3_COOH (= 60 Da), akin to that of aminopentol (**I**) (Fig SI1 – 3a), gives ion fragments *of m/z* 786, 726, 666, 606, 546 (Fig SI1 – 3; Table S1). Further structural information was obtained from the MS^3^ spectrum from fragmentation of the *m/z* 786 from MS^2^ (Fig SI1 – 3c). The ion fragment of *m/z* 744 indicates the loss of COCH_2_. The fragment at *m/z* 754 indicates neutral loss of CH_3_OH (= 32 Da), and is interpreted as indicating part of the terminal group and is again only a minor ion as seen for the novel pentafunctionalised compound. The fragment ion of *m/z* 471 indicates the neutral loss of 75 Da (after loss of all five regular acetylated hydroxyls) and indicates the full terminal group with the proposed structure -NHCOOCH_3_ (i.e. methylcarbamate). The ion fragment of *m/z* 231 is a common ion in BHP mass spectra (Talbot et al., 2003a,b). The ion of m/z 594 indicates neutral loss of the A+B ring fragment (loss of 192 Da) from the MS^3^ parent ion (m/z 786). This component has therefore been tentatively named 35-methylcarbamate-bacteriohopane-30,31,32,33,34-pentol (MC-pentol herein).

*
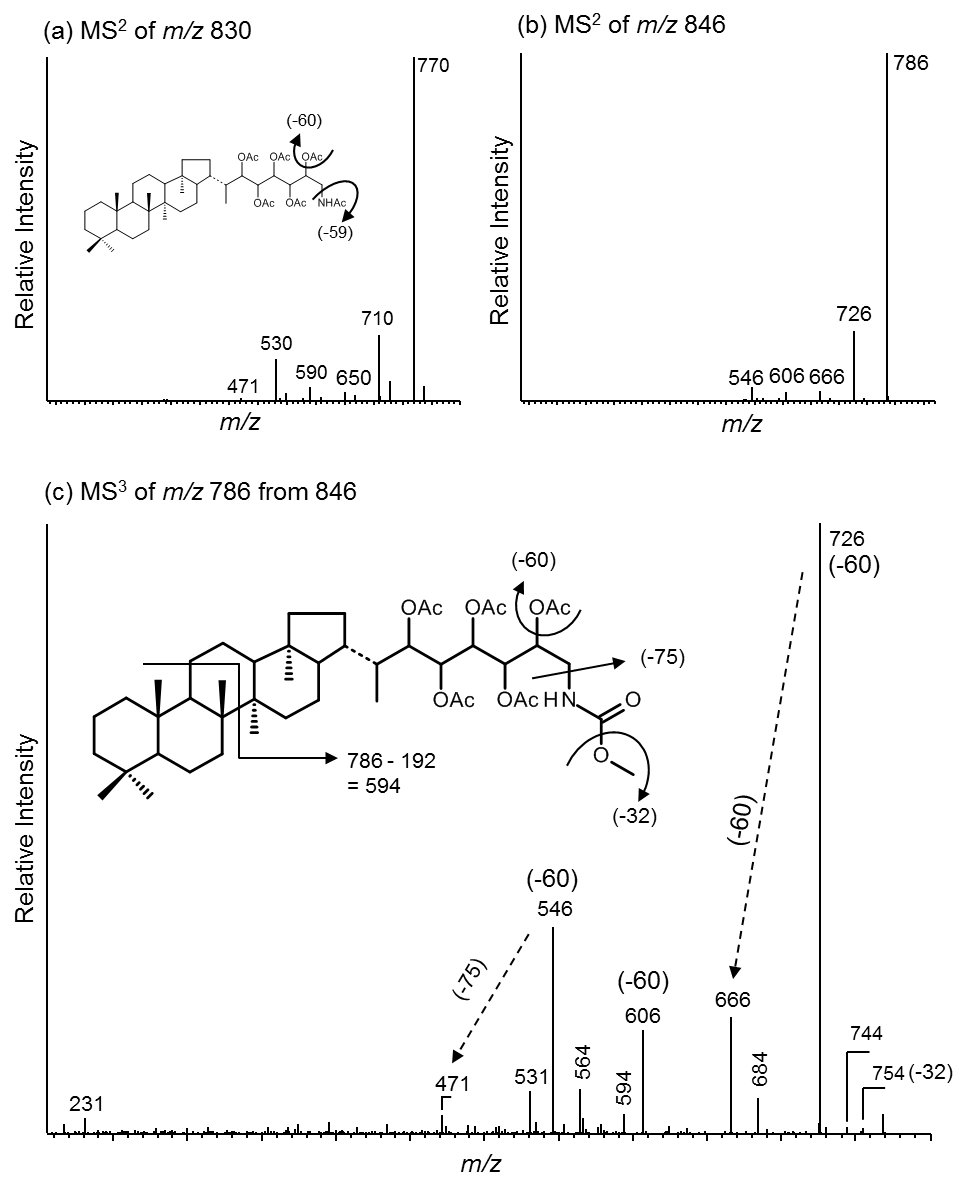
*

**Figure SI1 – 3.** (a) APCI MS^2^ spectrum of aminopentol (**I**); (b) APCI MS^2^ spectrum of proposed methylcarbamate-pentol (**I^MC^**); (c) APCI MS^3^ spectrum of **I^MC^**. Numbers in brackets indicate neutral molecule loss (Da). Ac = COCH_3_.

**Table S1 – 1**. Major fragment ions of aminopentol (**I**), aminopentol isomer (**I’**), aminotetrol (**II**), aminotriol (**III**), and their methylcarbamate (MC) homologues^a^.

|  | ‘Aminopentols’ | | | ‘Aminopentol isomers’ | | ‘Aminotetrols’ | | ‘Aminotriols’ | |
| --- | --- | --- | --- | --- | --- | --- | --- | --- | --- |
|  | | **I** | **I^MC^** | **I'**^b^ | **I^MC^'** | **II** | **II^MC^** | **III** | **III^MC^** |
| [M+H]^+^ | | **830** | **846** | **788** | **804** | **772** | **788** | **714** | **730** |
| [M+H-H_2_O]^+^ | |  |  | **770** | **786** |  |  |  |  |
| [M+H-CH_3_COOH]^+^ | | **770** | **786** | **728** | **744** | **712** | **728** | **654** | **670** |
| [M+H-CH_3_COOH-H_2_O]^+^ | |  |  | **710** | **726** |  |  |  |  |
| [M+H-CH_3_COOH-CH_3_OH]^+^ | |  | **754** |  | *712* |  | **696** |  | **638** |
| [M+H-CH_3_COOH-COCH_2_]^+^ | | **728** | **744** | *686* | *702* | **670** | **686** | **612** | **628** |
| [M+H-2CH_3_COOH]^+^ | | **710** | **726** | **668** | **684** | **652** | **668** | **594** | **610** |
| [M+H-2CH_3_COOH-H_2_O]^+^ | |  |  | **650** | **666** |  |  |  |  |
| [M+H-2CH_3_COOH-COCH_2_]^+^ | | **668** | **684** |  |  | **610** | **626** | *552* | *568* |
| [M+H-3CH_3_COOH]^+^ | | **650** | **666** | **608** | *624* | **592** | **608** | **534** | **550** |
| [M+H-3CH_3_COOH-H_2_O]^+^ | |  |  | **590** | **606** |  |  |  |  |
| [M+H-3CH_3_COOH-COCH_2_]^+^ | | **608** | **624** |  |  | **550** | **566** | *492* | *508* |
| [M+H-3CH_3_COOH-CH_3_CONH_2_]^+^ | |  |  |  |  |  |  | *475* |  |
| [M+H-4CH_3_COOH]^+^ | | **590** | **606** | **548** | *564* | **532** | 548 |  |  |
| [M+H-3CH_3_COOH-CH_3_OCONH_2_]^+^ | |  |  |  |  |  |  |  | **475** |
| [M+H-4CH_3_COOH-H_2_O]^+^ | |  |  | **530** | *546* |  |  |  |  |
| [M+H-4CH_3_COOH-COCH_2_]^+^ | | **548** | *564* |  |  | *490* | *506* |  |  |
| [M+H-4CH_3_COOH-CH_3_CONH_2_]^+^ | |  |  |  |  | *473* |  |  |  |
| [M+H-5CH_3_COOH]^+^ | | **530** | **546** |  |  |  |  |  |  |
| [M+H-4CH_3_COOH-CH_3_OCONH_2_]^+^ | |  |  |  |  |  | **473** |  |  |
| [M+H-4CH_3_COOH-H_2_O-CH_3_CONH_2_]^+^ | |  |  | **471** |  |  |  |  |  |
| [M+H-5CH_3_COOH-H_2_O]^+^ | |  |  |  |  |  |  |  |  |
| [M+H-4CH_3_COOH-H_2_O-CH_3_OCONH_2_]^+^ | |  |  |  | *471* |  |  |  |  |
| [M+H-5CH_3_COOH-COCH_2_]^+^ | |  |  |  |  |  |  |  |  |
| [M+H-5CH_3_COOH-CH_3_CONH_2_]^+^ | | **471** |  |  |  |  |  |  |  |
| [M+H-5CH_3_COOH-CH_3_OCONH_2_]^+^ | |  | **471** |  |  |  |  |  |  |

Bold text = observed ions;

italics = expected ion, not observed (cf. Talbot et al., 2003),

italics, underlined = not observed due to no MS^3^ spectrum for this compound.

^a^ Other minor fragment ions indicative of the hopanoid A+B rings, or their neutral molecule loss (-192 Da), are shown on Figures SI1 – 1, SI1 – 2, and SI1 – 3.

^b^ Aminopentol isomer (**I’**) was first described from a culture of the methanotroph *Methylovulm-*like strain 200 by van Winden et al. (2012). Although the mass spectrum indicated a hexafunctionalised BHP, the lower mass but earlier retention time coupled with the evidence of a free “OH” group (seen as loss of water in the APCI MS^2^ spectrum) suggested an aminopentol structure in which one of the OH groups has not acetylated, potentially due to steric hindrance if 2 OH groups are attached to the same C atom (van Winden et al., 2012).

**References**

Cvejic JH, Bodrossy L, Kovacs KL, Rohmer M (2000) Bacterial triterpenoids of the hopane series from the methanotrophic bacteria Methylocaldum spp.: phylogenetic implications and first evidence for an unsaturated aminobacteriopanepolyol. FEMS Microbiol Lett 182: 361-365.

Talbot HM, Watson DF, Murrell JC, Carter JF, Farrimond P (2001) Analysis of intact bacteriohopanepolyols from methanotrophic bacteria by reversed-phase high-performance liquid chromatography-atmospheric pressure chemical ionisation mass spectrometry. J Chromatogr A 921: 175-185.

Talbot HM, Squier AH, Keely BJ, Farrimond P, (2003a) Atmospheric pressure chemical ionisation reversed-phase liquid chromatography/ion trap mass spectrometry of intact bacteriohopanepolyols. Rapid Commun Mass Spectrom 17: 728-737.

Talbot HM, Summons R, Jahnke L, Farrimond P, (2003b) Characteristic fragmentation of bacteriohopanepolyols during atmospheric pressure chemical ionisation liquid chromatography/ion trap mass spectrometry. Rapid Commun Mass Spectrom 17: 2788-2796.

Talbot HM, Farrimond P, Schaeffer P, Pancost RD, (2005) Bacteriohopanepolyols in hydrothermal vent biogenic silicates. Org Geochem 36: 663-672.

Talbot HM, Rohmer M, Farrimond P (2007a) Rapid structural elucidation of composite bacterial hopanoids by atmospheric pressure chemical ionisation liquid chromatography/ion trap mass spectrometry. Rapid Commun Mass Spectrom 21: 880-892.

Talbot HM, Rohmer M, Farrimond P, (2007b) Structural characterisation of unsaturated bacterial hopanoids by atmospheric pressure chemical ionisation liquid chromatography/ion trap mass spectrometry. Rapid Commun Mass Spectrom 21: 1613-1622.

Talbot HM, Summons RE, Jahnke LL, Cockell CS, Rohmer M, Farrimond P (2008) Cyanobacterial bacteriohopanepolyol signatures from cultures and natural environmental settings. Org Geochem 39: 232-263.

Talbot HM, Bischoff J, Inglis GN, Collinson ME, Pancost RD (2016) Polyfunctionalised bio- and geohopanoids in the Eocene Cobham Lignite. Org Geochem 96: 77-92.

van Winden JF, Talbot HM, Kip N, Reichart GJ, Pol A, McNamara NP, et al. (2012) Bacteriohopanepolyol signatures as markers for methanotrophic bacteria in peat moss. Geochim Cosmochim Acta 77: 52-61.
